# Diffusion-weighted magnetic resonance imaging in acute reversible toxic leukoencephalopathy: A report of two cases

**DOI:** 10.4103/0971-3026.69354

**Published:** 2010-08

**Authors:** S Sivasubramanian, Srikant Moorthy, KP Sreekumar, R Rajesh Kannan

**Affiliations:** Department of Radiology, Amrita Institute of Medical Sciences, Elamakkara, Cochin – 682 041, India

**Keywords:** 5-FU, diffusion restriction, leukoencephalopathy, uremia

## Abstract

Acute toxic leukoencephalopathy may be caused by endogenous or exogenous toxins. It may reverse clinically if the offending agent is withdrawn or the underlying condition is treated. However, demonstration of reversibility on imaging, especially with diffusion-weighted MRI, has been reported only very recently. We report two such cases.

## Introduction

Toxic leukoencephalopathies are sometimes known to reverse clinically if the offending agent is arrested before irreversible damage occurs.[1,2] However, an imaging correlate of this reversal has been reported only recently.[2] We present two cases of acute toxic leukencephalopathies due to different etiologies, both presenting with identical diffusion-weighted MRI features. Both patients had an acute onset of neurological deficit but recovered within 2 to 3 days. MRI after recovery showed resolution of the findings.

## Case Reports

### Case 1

A 45-year-old man, known to have systemic hypertension on irregular medication, presented with periorbital puffiness and sudden onset of slurring of speech, followed by progressive weakness of the left side of the body.

On examination, the patient was conscious and well oriented. The blood pressure was elevated (182/108 mm Hg). Facial deviation to the left was present and decreased power was noted in the left upper limb and lower limb (power 3/5 and 3/5, respectively). Blood investigations revealed a BUN (blood urea nitrogen) of 185.7 mg/dl and a serum creatinine of 8.4 mg/dl. Serum sodium was within normal limits. The patient underwent hemodialysis three times and had resolution of the neurological findings immediately after dialysis.

**Figure 1 (A-D):**
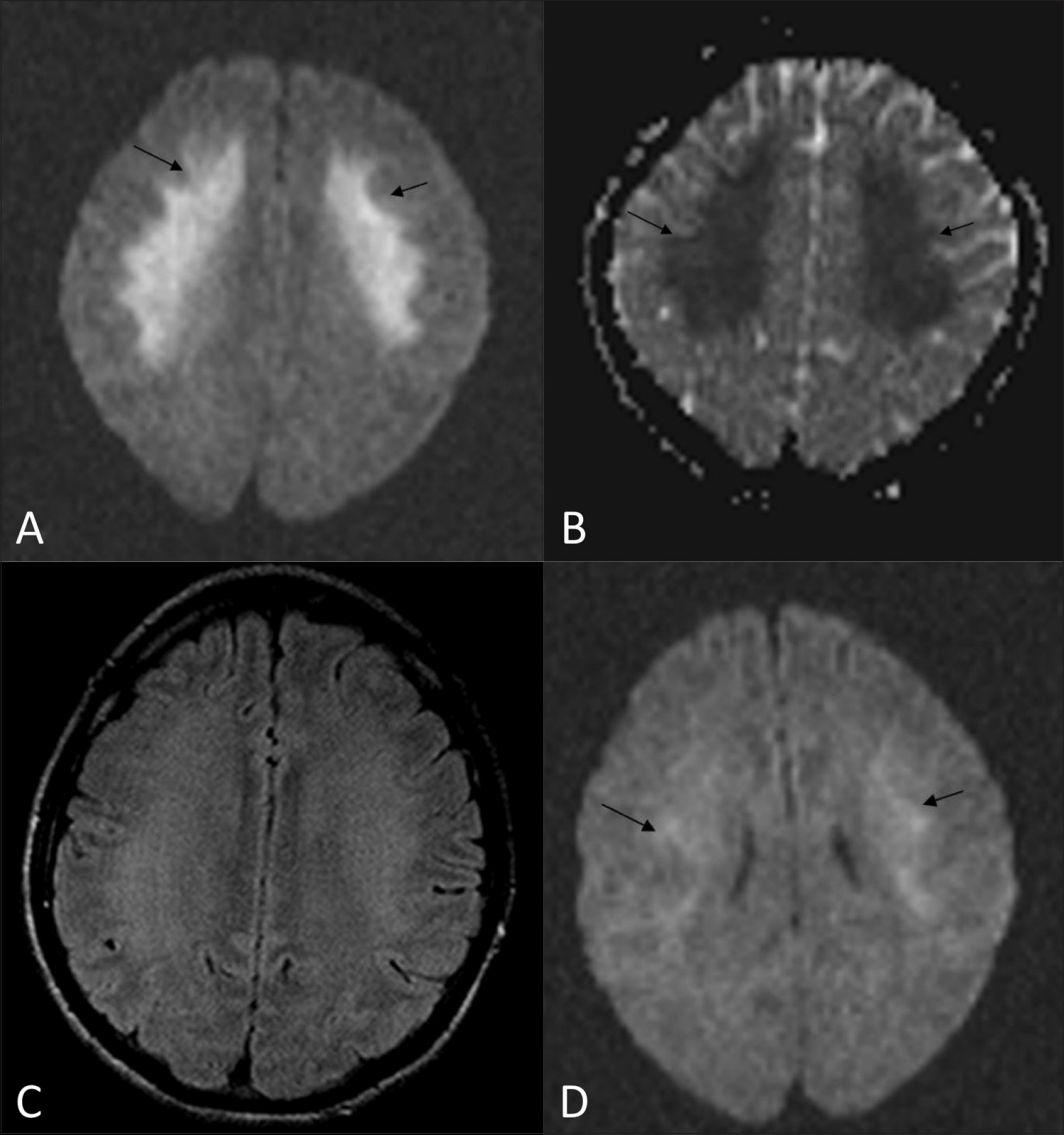
MRI. Axial diffusion-weighted (b1000) image (A) shows significant restriction of diffusion (arrows) in the frontal lobe white matter. The corresponding ADC image (B) shows decreased ADC, as evident by the dark signal (arrows) in the region of reduced diffusion (ADC: 350 – 380 10^-3^ mm^2^/s). Axial FLAIR image (C) shows no significant changes in the region of reduced diffusion. Follow-up MRI (diffusion-weighted imaging) after 3 days (D) shows resolution of the white matter changes

MRI during the acute episode revealed significant symmetrical diffusion restriction [Figure 1A], with reduced apparent diffusion coefficient (ADC) values [Figure 1B] in the frontal white matter bilaterally (in the range of 350 – 380 in the involved white matter, compared with 800 – 950 in the unaffected white matter). Fluid-attenuated inversion recovery (FLAIR) images [Figure 1C] showed only subtle signal changes. There was complete resolution on the follow-up MRI after 3 days [Figure 1D]. No abnormal enhancement was seen.

### Case 2

A 62-year-old man who was recently diagnosed with rectal carcinoma was started on neoadjuvant chemotherapy with 5-fluouracil (5-FU) and leucovorin. About 4 days after starting chemotherapy, the patient presented to the emergency department with acute onset of aphasia. No other neurological deficits were noted. The 5-FU was stopped, and the patient recovered completely within 6 to 8 hours.

**Figure 2 (A-D):**
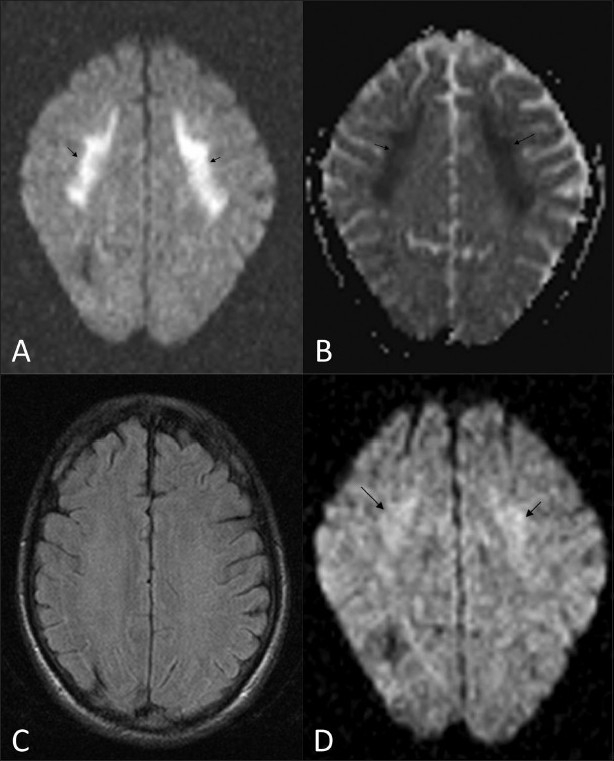
MRI. Axial diffusion-weighted (b1000) image (A) shows significant restriction of diffusion (arrows) in the frontal lobe white matter and the splenium of the corpus callosum (not shown). Corresponding ADC image (B) shows decreased ADC, as evident by the dark signal (arrows) in the region of reduced diffusion (ADC: 350 – 400 10^-3^ mm^2^/s). Axial FLAIR image (C) shows no significant changes in the region of reduced diffusion. Follow-up MRI (diffusionweighted imaging) (D) shows resolution of the white matter changes (ADC: 650 – 700 10^-3^ mm^2^/s)

MRI during the episode showed significant diffusion restriction [Figure 2A], with reduced ADC values [Figure 2B] in the frontal white matter bilaterally and the splenium of the corpus callosum (350 – 400 in the involved white matter, compared with 800 – 950 in the unaffected white matter). FLAIR images showed no signal changes [Figure 2C]. No abnormal enhancement was seen. Significant resolution of the findings was seen on the diffusion-weighted images on an MRI done after 3 days (ADC values were in the range of 650 – 700) [Figure 2D].

## Discussion

Toxic leukoencephalopathy[1] is a term used to denote the damage to cerebral white matter caused by many agents, e.g., chemotherapeutic agents, immunosuppressive therapy, antimicrobial agents, environmental toxins, drug abuse, organ failure (liver/kidneys), etc. The drugs[2] that are known to cause toxic encephalopathy include chemotherapeutic agents such as methotrexate, 5-FU, fludarabine, tacrolimus, and cyclosporine. Metronidazole, an antimicrobial agent has also been reported to cause toxic leukoencephalopathy.[2]

The neurological findings in acute toxic leukoencephalopathy can be reversed if promptly recognized and treated. Recent reports[2] have highlighted the value of diffusion-weighted imaging (DWI) to document radiological reversal of findings in these patients. Hence, we have referred to this entity as ‘acute reversible toxic leukoencephalopathy.’

In our first patient, the cause of the imaging findings was uremia which is known to cause white matter damage.

Although this patient had elevated blood pressure, the MRI findings were not typical of posterior reversible encephalopathy syndrome (PRES)[2–4] in which there is parieto-occipital distribution of lesions, mainly involving the cortex and the subcortical white matter. Periventricular white matter involvement and diffusion restriction are rare and are described as atypical manifestations in PRES. PRES is usually known to occur following sudden elevation of blood pressure. Some cases with a history of exposure to toxins, which could not be classified as PRES were previously labelled as atypical PRES.[5]

Our patient recovered after dialysis. The diffusion restriction during the acute episode was probably due to uremic toxins,[4] which have been described to cause injury to the white matter. There are a few recent reports of reversible diffusion restriction of white matter in patients with uremia, the imaging findings being postulated to be due to cytotoxic edema.[6,7] However, the mechanisms of white matter damage in uremia are not clearly known, though it is likely that they are similar to those that occur with other toxins.

In our second patient, the cause was 5-FU, because stopping the drug led to a full recovery. Similar reports of 5-FU–induced encephalopathy have been reported earlier in literature.[8,9]

Mckinney *et al*. has recently reported 19 cases of toxic leukoencephalopathy, most of them related to chemotherapeutic drugs.[2] The regions commonly affected were the white matter of the frontal, parietal, or occipital lobes and the corpus callosum. The basal ganglia, thalami, and internal capsule were only rarely involved.[2]

The cause of diffusion restriction in toxic encephalopathy[2] is not exactly known, but is presumably due to either intramyelinic edema (myelin vacuolation), cytotoxicity due to endothelial damage, or due to direct toxic demyelination.[2]

The bilateral distribution and the pattern on DWI can be mistaken for ischemia if the radiologist is not aware of the possibility of toxic leukencephalopathy. Prompt recognition and appropriate management may reverse the neurological manifestation.

A larger series of patients needs to be studied to assess the specificity of these findings to diagnose toxic leukoencephalopathy.

## Conclusion

In patients who clinically present with acute toxic leukoencephalopathy, DWI plays a significant role, both during the acute episode and during follow-up, to demonstrate reversibility after therapy.

The radiologist should be aware of this condition and its imaging manifestations, as well as of the potential reversibility. Early identification can lead to prompt and appropriate management.
